# NF-κB-dependent repression of Sox18 transcription factor requires the epigenetic regulators histone deacetylases 1 and 2 in acute lung injury

**DOI:** 10.3389/fphys.2022.947537

**Published:** 2022-08-04

**Authors:** Evgeny A. Zemskov, Christine M. Gross, Saurabh Aggarwal, Marina A. Zemskova, Xiaomin Wu, Chenxin Gu, Ting Wang, Haiyang Tang, Stephen M. Black

**Affiliations:** ^1^ Center for Translational Science, Florida International University, Port St. Lucie, FL, United States; ^2^ Department of Cellular Biology and Pharmacology, Herbert Wertheim College of Medicine, Florida International University, Miami, FL, United States; ^3^ Department of Medicine at Broward Health Medical Center, Fort Lauderdale, FL, United States; ^4^ Department of Anesthesiology, The University of Alabama, Birmingham, AL, United States; ^5^ Department of Medicine, The University of Arizona Health Sciences, Tucson, AZ, United States; ^6^ College of Veterinary Medicine, Northwest A&F University, Xianyang, China; ^7^ State Key Laboratory of Respiratory Disease, Guangzhou Institute of Respiratory Disease, First Affiliated Hospital of Guangzhou Medical University, Guangzhou, China; ^8^ Department of Environmental Health Sciences, Robert Stempel College of Public Health and Social Work, Florida International University, Miami, FL, United States

**Keywords:** transcriptional repression, histone deacetylases, acute lung injury, sepsis, epigenetics

## Abstract

In acute lung injury (ALI), the NF-κB-mediated downregulation of Sox18 gene expression leads to the disruption of the pulmonary endothelial barrier. Previous studies have suggested that the action of NF-κB as a transcriptional repressor also requires the action of class I histone deacetylases (HDACs). Thus, the purpose of this study was to investigate and further delineate the mechanism of Sox18 repression during lipopolysaccharide (LPS) induced ALI. Using selective inhibitors and specific siRNA-driven depletion of HDACs 1-3 in human lung microvascular endothelial cells (HLMVEC) we were able to demonstrate a critical role for HDACs 1 and 2 in the LPS-mediated repression of Sox18 gene expression and the loss of endothelial monolayer integrity. Moreover, our data demonstrate that HDAC1 associates with a transcription-repressive complex within the NF-κB-binding site of Sox18 promoter. Further, we were able to show that the selective inhibitor of HDAC1, tacedinaline, significantly reduced the endothelial permeability and injury associated with LPS challenge in the mouse lung. Taken together, our data demonstrate, for the first time, that transcription repressors HDACs 1 and 2 are involved in pathological mechanism of ALI and can be considered as therapeutic targets.

## Introduction

Hyperpermeability of the pulmonary endothelium is a hallmark of acute lung injury and acute respiratory distress syndrome (ALI/ARDS) (Maniatis et al., 2008). To date, no effective treatment to protect or restore the endothelial barrier function in ALI/ARDS patients has been established. Therefore, studies investigating the molecular mechanisms of endothelial hyperpermeability associated with ALI/ARDS are necessary to drive new therapeutic advances. Sox18 is a transcription factor critical for maintaining endothelial barrier function (Gross et al., 2014; Gross et al., 2018) and we have previously shown a functional link between the downregulation of Sox18 expression and the loss of barrier function in HLMVEC challenged with lipopolysaccharide (LPS) (Gross et al., 2018). Repression of Sox18 expression is followed by the downregulation of Claudin-5, tight junction (TJ) protein which is intimately involved in the regulation of the endothelial barrier function (Nitta et al., 2003; Fontijn et al., 2008). Under conditions of increased shear stress, Sox18 and Claudin-5 are upregulated in EC; siRNA-mediated depletion of Sox18 causes EC monolayer integrity loss in the transendothelial electrical resistance (TER) assay (Gross et al., 2014). This is associated with a decrease in Claudin-5 levels (Gross et al., 2014). The effect of SOX18 appears to be specific to Claudin-5 as other Claudins were not affected by Sox18 depletion (Gross et al., 2014). However, siRNA-mediated depletion of Claudin-5 alone led to ZO-1 and VE-cadherin loss and barrier dysfunction, whereas Claudin-5 over-expression reversed the effect of Claudin-5 depletion on EC monolayer integrity (Gross et al., 2014). Overexpression of Sox18 prevents the downregulation of Claudin-5 and protects the endothelial barrier function *in vitro* and *in vivo* (Fontijn et al., 2008; Gross et al., 2014; Gross et al., 2018) clearly demonstrating a critical role of Sox18/Claudin-5 axis in the endothelial integrity.

The transcription factor, NF-κB is one of the most well-established inducers of the inflammatory response and is activated by a wide spectrum of stimuli (Pahl, 1999; Fiordelisi et al., 2019). NFκB-dependent activation of pro-inflammatory cytokines/chemokines expression is well documented, and this plays an important role in the inflammatory pathway that leads to the injury associated with ALI/ARDS (Schwartz et al., 1996; Baetz et al., 2005). However, recent studies have also revealed that activated NF-κB can repress the transcription of certain genes (Kouba et al., 1999; Campbell et al., 2004; Shaw et al., 2006). Our recent work has shown that the loss of Sox18 expression in ALI is NF-κB-dependent and identified the NF-κB-binding site responsible (Gross et al., 2018). Prior work has suggested that NF-κB-mediated transcriptional repression is associated with the formation of multiprotein complexes consisting of NF-κB subunits and class I histone deacetylases (HDACs), HDACs 1, 2, or 3 (Campbell et al., 2004; Shaw et al., 2006). Available data suggest that the p65 (RelA) subunit of NF-κB can recruit HDACs 1 and 2 to the chromatin where the latter can function as transcription co-repressors (Ashburner et al., 2001). However, to date, an existence of such repressive complex essential for ALI/ARDS pathology has been not explored. Thus, to further elucidate the mechanism of NF-κB-dependent repression of Sox18 gene expression, we studied the potential involvement of the class I HDACs in this process.

Using several lines of evidence, we show here that nuclear HDACs 1 and 2 are critically involved in the mechanism of NF-κB-mediated repression of Sox18 gene expression during ALI. Furthermore, we were able to demonstrate significant protective effects using the selective HDAC1 inhibitor, tacedinaline, in a mouse model of LPS-mediated ALI. Taken together, our data reveal an involvement of HDACs 1 and 2 in NF-κB-dependent transcription repression of Sox18 and suggest a potential therapeutic application of HDAC1/2 inhibitors in ALI/ARDS.

## Materials and methods

### Materials

Class I HDAC inhibitors, tacedinaline (Cat # 2952), FK228 (Cat # 3515), MI192 (Cat # 5647), and RGFP966 (Cat # 6728) were obtained from Tocris (Minneapolis, MN). The mouse monoclonal anti-HDAC1 and anti-HDAC2 antibodies (Cat ## 17-608 and 17-10237), and the Chromatin Immunoprecipitation assay kit (Cat # 17-295) were from EMD Biosciences (Philadelphia, PA). The mouse monoclonal anti-Sox18 antibody (Cat # sc-166025) was obtained from Santa Cruz Technology (Santa Cruz, CA). The rabbit monoclonal anti-acetyl-Lys27 histone H3 antibody (Cat # 8173), the rabbit monoclonal anti-phospho-Ser536 NF-κB p65 antibody (Cat # 3033), the rabbit monoclonal anti-acetyl-Lys310 NF-κB p65 (Cat # 12629) and the rabbit polyclonal anti-acetyl-Lys40 *a*-tubulin antibody (Cat # 3971) were from Cell Signaling (Danvers, MA). The mouse monoclonal anti-β-actin antibody (Cat # A3854) and *E. coli* endotoxin, LPS (Cat #L3129), were obtained from Sigma-Aldrich (St. Louis, MO). HDAC1- and HDAC2-specific siRNAs (Cat ## sc-29343 and sc-29345) were obtained from Santa Cruz Technology (Santa Cruz, CA). Universal blocking reagent, Power Block (Cat # HK085) was obtained from BioGenex (Fremont, CA). Effectene (Cat # 301425) and HiPerFect (Cat # 301704) transfection reagents were obtained from Qiagen (Valencia, CA). The pGL3-luciferase Reporter Vector (Cat #E1751), *ß*-galactosidase enzyme assay (Cat #E2000) and Luciferase Assay System (Cat #E1500) were from Promega Corporation (Madison, WI).

### Cell culture

Human lung microvascular cells (HLMVEC) (Cat # CC-2527) were obtained from Lonza (Walkersville, MD) and grown in EBM-2 cell culture medium supplemented with respective bullet kit (Cat # CC-3202) (Lonza) at 37°C in a humidified atmosphere of 5% CO_2_. The cells were utilized between passages 5 to 7. To inhibit HDACs, the cells were pre-treated with respective inhibitors for 30 min. *E. coli* LPS (Sigma-Aldrich) was used to challenge HLMVEC at 1-2 EU/ml. For HDAC silencing, HLMVEC were transfected with 20–100 nM HDAC1-, or HDAC2- specific small interfering RNA (siRNA) duplexes (Santa Cruz Biotechnology) using HiPerFect transfection reagent, according to the manufacturer’s instructions. A scrambled siRNA was used as a negative control. Validation of the gene silencing effect was performed using immunoblotting analysis. For plasmid transfections, Effectene transfection reagent was used according to the manufacturer’s instructions.

### Promoter activity assay

The first 1600 bp of the Sox18 promoter were subcloned into a pGL3 luciferase reporter vector (Promega, Madison, WI). To determine the mechanism of NF-κB-mediated repression of the Sox18 promoter, HLMVEC were simultaneously co-transfected with either HDAC1 or HDAC2 siRNA along with the Sox18 luciferase promoter plasmid and *ß*-galactosidase plasmid on 6-well plates for 48 h followed by LPS exposure (1 EU/ml, 4 h). In addition, HLMVEC transfected with the Sox18 promoter construct and *ß*-galactosidase for 48 h were treated for 30 min With either the HDAC1 inhibitor, Tacedinaline (150 μM), or the HDAC2 inhibitor, FK228 (10 nM), and then exposed to LPS (1 EU/ml, 4 h). The luciferase activity in HLMVEC lysates was determined using the Luciferase assay system (Promega), as described previously (Kumar et al., 2008). As a control of transfection efficiency, *ß*-galactosidase activity was measured using the *ß*-galactosidase enzyme assay (Promega).

### SDS-PAGE and immunoblotting

Lysates obtained from HLMVEC or lung tissues were subjected to SDS-PAGE in 4–20% Tris-glycine gels (Cat # 4561094) (Bio-Rad, Hercules, CA) followed by electrotransfer to PVDF membranes (Cat # 1704156) (Bio-Rad). Specific protein bands were detected using respective primary and secondary antibodies and visualized using Super Signal West Femto Chemiluminescence kit (Cat # PI34095) (Fisher Scientific, Hampton, NH) using LI-COR Odyssey image station (Lincoln, NE). Specific protein images obtained were quantified using LI-COR Image Station software. Protein expressions were normalized by re-probing membranes with an anti-β-actin antibody (Sigma-Aldrich).

### Transendothelial electrical resistance assay

TER assays were performed to measure HLMVEC integrity using ECIS instrument Z-Theta model (Applied BioPhysics, Troy, NY) as described previously (Garcia et al., 2000). The cells were grown in 8-well 8W10E ECIS arrays (Applied BioPhysics) to 100%-confluency in complete EGM-2 medium. The cells were pre-treated with HDAC inhibitors or vehicle as a control for 30 min, then the cells were challenged with LPS, and TER response changes were real-time recorded. Initial resistance in all array wells was normalized to 1.

### Chromatin immunoprecipitation assay

Chromatin immunoprecipitation (ChIP) was performed using HLMVEC (∼1.0 × 10^6^) cultured in 10-cm Petri dishes using the ChIP Assay Kit (EMD Biosciences), as previously described (Kumar et al., 2008). The cells were treated with 1% formaldehyde for 10 min at 37°C to cross-link DNA-protein complexes. The cell lysates were sonicated to obtain DNA fragments approx. 200-1,000 bp. The supernatants were incubated overnight at 4°C with an anti-HDAC1 antibody (4 µg/sample), or an anti-HDAC2 antibody (2 µg/sample). As a negative control, normal mouse IgG were used accordingly. The DNA was precipitated with ethanol and resuspended in water. DNA concentrations were measured and equal DNA amounts were used for PCR with HotStarTaq DNA Polymerase (Cat # 203203) (Qiagen). The NF-κB binding site 1 in the human Sox18 promoter is located −1082–1073 bp upstream of the transcriptional start site. The primers used to amplify NF-κB-binding site 1 in the Sox18 promoter were 5′-caa​gac​ctg​tgg​cct​cta​cc-3’ (forward) and 5′-ctg​agg​gtc​tcc​ctc​tgg​a-3’ (reverse) to obtain a fragment of 200 bp. As a positive control, DNA obtained from total lysates was used.

### Real-time RT-PCR

Total RNA was isolated from HLMVEC using QIAshredder (Cat # 79654) (Qiagen) and the RNeasy Mini Kit (Cat # 74104) (Qiagen). cDNA was prepared from 2 μg of total RNA using SuperScript VILO Master Mix (Cat # 11755050) according to the manufacturer’s protocol (Fisher Scientific). cDNA samples diluted with water (1:10) were used for qPCR using a QuantiTect SYBR Green PCR Kit (Cat # 204143) (Qiagen) in a QuantStudio 3 System (Applied Biosystems, Waltham, MA). Data were analyzed by the 2^−ΔΔCt^ method. As a reference, expression of *β*_2_-microglobulin was used.

### Animals

Non-pyrogenic male C57BL/6 mice aged 6–8 weeks were kept in a room with tightly controlled light hours (12/12 h of light/dark cycle), appropriate temperature (22–24°C) and humidity range (55 ± 5%). Mice had full access to standard laboratory water and diet. All animal research was authorized by the institutional animal care and use committees at Florida International University, the University of Arizona as well as Northwest A&F University, and also confirmed with the Animal Welfare Act. The methodologies used in this investigation followed the approved guidelines.

### LPS model of ALI

Age- and weight-matched male mice (*n* = 20) were sedated with a ketamine/xylazine admixture (100/2 mg/kg) intramuscular injection and randomly divided into four study groups: control, tacedinaline alone, LPS alone, and LPS + tacedinaline (*n* = 5 per group). LPS (2 mg/kg in 50 μL saline) was injected intratracheally to cause ALI. Mice in the control and tacedinaline groups were injected intraperitoneally with saline and tacedinaline (5 mg/kg), respectively, 1 h before LPS treatment. Mice were sacrificed 6 h after receiving LPS. For further investigation, lung tissues and BALF were taken.

### Measurement of leukocyte infiltration and protein concentration in BALF

The lungs were lavaged with 1 ml of PBS using a tracheal cannula, and then BALF samples were taken. To pellet the cells, BALF samples were centrifuged at 500 g for 10 min at 4°C. A standard haemocytometer was used to count total leukocytes in BALF. A BCA protein assay kit was used to measure total protein contents in BALF.

### Immunohistochemical analysis of the mouse lung

Prior to tissue preparation with paraffin-embedded blocks, lungs were perfused with 10% formalin at 15 cm H_2_O pressure and submerged in the same solution, and then 4 μm sections were cut and stained with hematoxylin and eosin (H&E). Histopathological evaluation was completed by two investigators who were not aware of the details of the grouping. The presence of neutrophils, hyaline membranes and proteinaceous debris in the alveolar and interstitial space, as well as thickening of the alveolar septal, were all scored on H&E stained sections, as stated previously (Matute-Bello et al., 2011).

### Myeloperoxidase staining

Mouse lungs were embedded in paraffin and sectioned, then placed on treated glass slides (Superfrost plus, Cat # 12-550–15; Fisher Scientific) and air dried overnight. The slides were exposed to an oven at 60°C for 30 min on alternate days, then de-paraffinized with xylene and infiltrated sequentially by gradually decreasing concentrations of ethanol and finally into distilled water. Endogenous peroxidase was inhibited with 0.3% H_2_O_2_ (5 min) before being cleansed twice with distilled water. Slides were preprocessed with citrate (pH 6), rinsed in distilled water, incubated in Power Block (BioGenex), and rinsed in distilled water again, and then deposited in 1x PBS for 5 min, followed by incubation in anti-myeloperoxidase (MPO) antibody (1:200 dilution, Cat # ab139748, Abcam, Cambridge, MA) for 30 min at RT. The slides were cleansed twice in PBS before being incubated for 30 min with a secondary peroxidase-labeled goat anti-rabbit IgG. Diaminobenzidine (Cat #K3468, DAB + substrate kit, Dako Corp.) was used to identify bound antibody. As a counterstain, hematoxylin was utilized. The existence of neutrophils within the alveolar and interstitial spaces was scored on MPO-stained slides, as described previously (Rafikov et al., 2014).

### Statistical analysis

GraphPad Prism software was used for statistical analysis. Data presented as means ± SEM. Statistical significance was analyzed by the unpaired *t*-test (for 2 groups) or ANOVA (for ≥3 groups) with Newman-Keuls post-hoc test. P< 0.05 was considered as significant.

## Results

### Inhibition of HDAC1 or HDAC2 preserves monolayer integrity in LPS-challenged human lung microvascular endothelial cells

**FIGURE 1:**
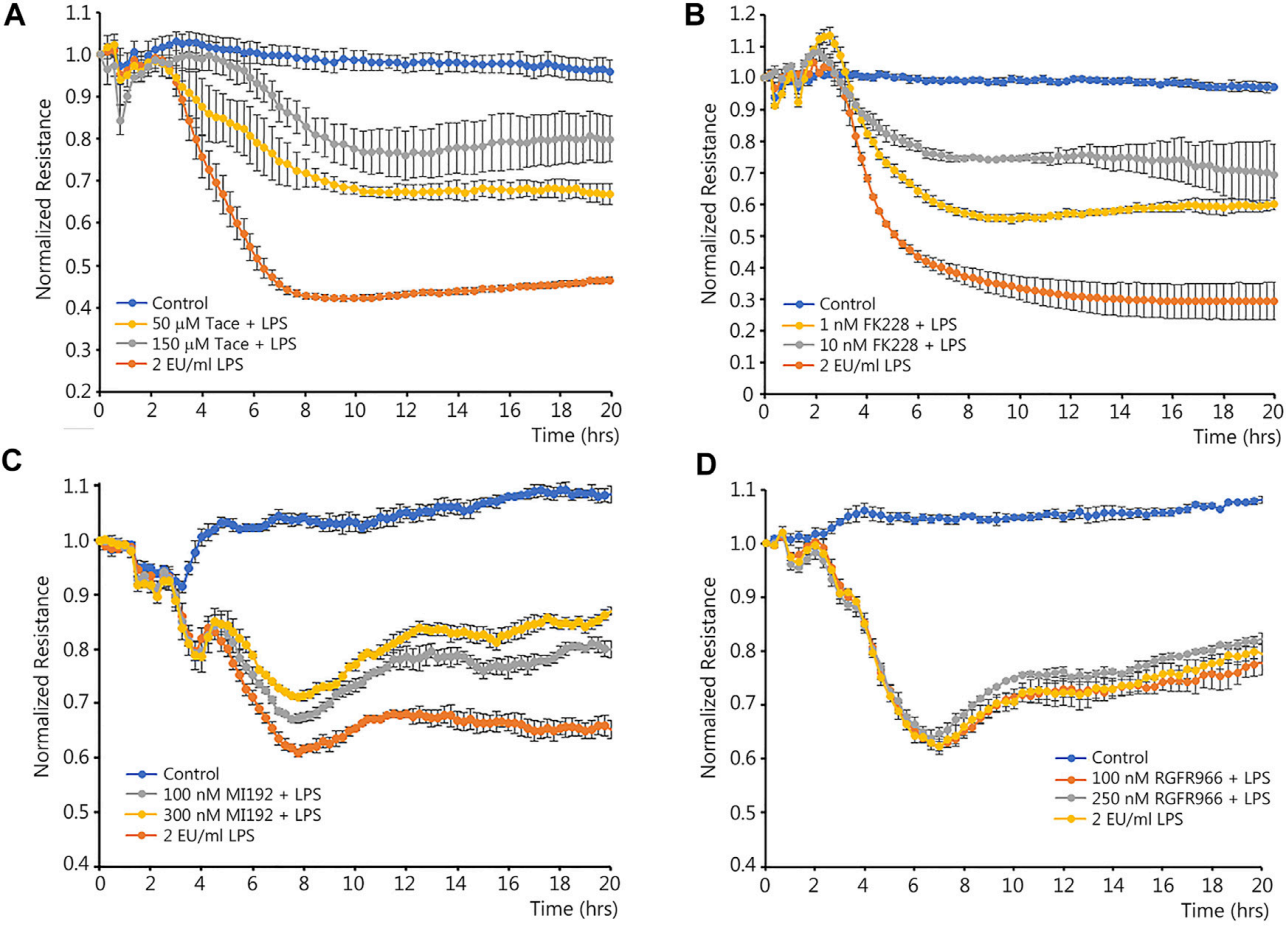
Effects of class I HDACs selective inhibitors on LPS-induced endothelial barrier disruption in cultured human lung microvascular endothelial cells. HLMVEC monolayers grown in ECIS arrays were pre-treated with HDAC inhibitors or vehicle for 30 min then challenged with LPS. TER was recorded in real time to evaluate possible barrier-protective effects of HDAC inhibitors. Data are shown for tacedinaline (HDAC -1 and -3 inhibitor) **(A)**, FK228 (HDAC -1 and -2 inhibitor) **(B)**, MI192 (HDAC -2 and -3 inhibitor) **(C)**, and RGFP966 (HDAC3 inhibitor) **(D)**. Data are mean ± SEM, *n* = 4. *p < 0.05 *vs*. Control; †*p* < 0.05 *vs*. LPS alone.

Class I HDACs have been associated with the NF-κB-mediated downregulation of certain genes (Campbell et al., 2004; Shaw et al., 2006). As we have previously shown that LPS-mediated activation of NF-κB leads to the downregulation of Sox18 (Gross et al., 2018), we initially investigated if selective inhibitors of HDAC1-3 could reduce the barrier disruption in LPS-challenged HLMVEC. Changes in the barrier integrity were recorded in real time using a transendothelial electrical resistance (TER) assay in HLMVEC challenged with LPS (Figure 1). Analysis of the TER data obtained using inhibitors for HDAC1/3 (Figure 1A), HDAC1/2 (Figure 1B), HDAC2/3 (Figure 1C) and HDAC3 (Figure 1D) demonstrated a significant barrier-protective effect when HDAC -1 or -2 were inhibited (Figures 1A–C), while HDAC3 inhibition did not protect against LPS-induced barrier disruption (Figure 1D).

### HDACs 1 and 2 are involved in the LPS-mediated down-regulation of Sox18 expression in human lung microvascular endothelial cells

**FIGURE 2:**
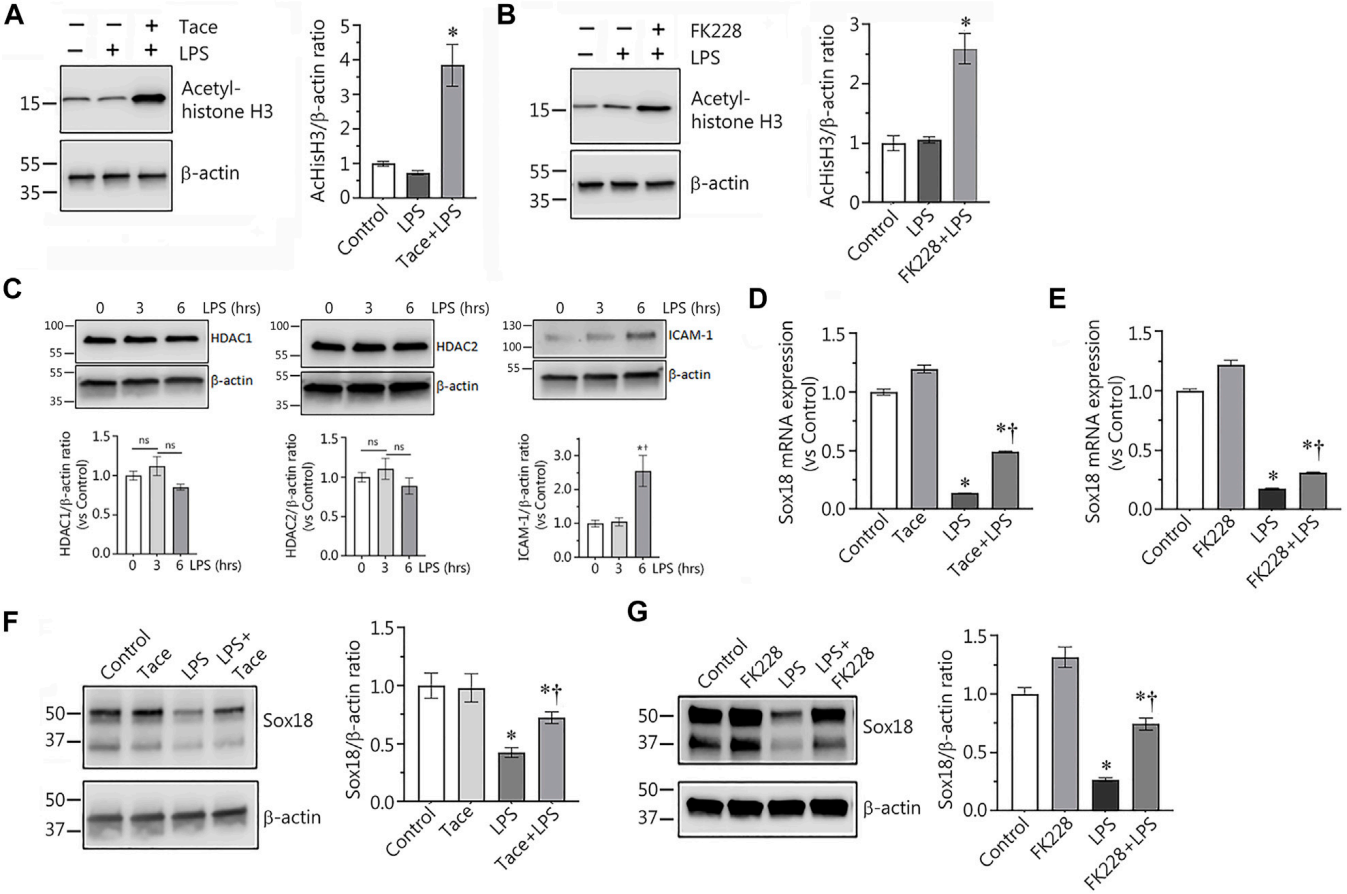
Selective inhibitors of HDAC1 and HDAC2 significantly protect expression of Sox18 at mRNA and protein levels in LPS-challenged in cultured human lung microvascular endothelial cells. In HLMVEC, pharmacological inhibition of HDACs 1 or 2 leads to accumulation of acetylated histone H3 demonstrating specific effects of the inhibitors **(A,B)**. LPS challenge affected neither HDAC1 nor HDAC2 protein levels, however, ICAM-1 expression is significantly increased **(C)**. At the same time, the inhibitors significantly attenuate the LPS-induced repression of Sox18 mRNA expression **(D,E)** and Sox18 protein levels **(F,G)**. The cells were pre-treated with the inhibitors or vehicle for 30 min then challenged with 2 EU/ml LPS for 6 h. Data are mean ± SEM, *n* = 3–6. *p < 0.05 *vs*. Control; †*p* < 0.05 *vs*. LPS alone.

Using immunoblot analysis with an antibody against acetylated histone H3 we confirmed that the HDAC 1 and 2 inhibitors, tacedinaline and FK228, significantly increased the levels of acetylated histone H3, a HDAC1/2 specific protein substrate, demonstrating the efficacy of the inhibitors in preventing HDAC1/2-dependent histone deacetylation (Figures 2A,B). LPS exposure did not change HDAC1/2 protein levels, but increased the expression of the endothelial inflammatory receptor, ICAM-1 (Figure 2C). Next, we evaluated the levels of Sox18 mRNA in LPS-challenged EC in the presence or absence of these inhibitors to test the possible role of HDACs 1 and 2 in the downregulation of Sox18 gene expression. Our data clearly indicate that LPS treatment repressed Sox18 mRNA expression, however, pre-treatment with either tacedinaline or FK228 attenuated the LPS-mediated decrease in SOX18 mRNA levels (Figures 2D,E). Similarly, pre-treatment with either the HDAC1 or HDAC2 inhibitor significantly protected Sox18 protein levels in LPS-treated HLMVEC (Figures 2F,G). Together these data suggest that HDAC1/2 are involved in the LPS-mediated decrease in Sox18 expression.

### HDAC1 or HDAC2 inhibition does not affect LPS-mediated activation of NF-κB in human lung microvascular endothelial cells

**FIGURE 3:**
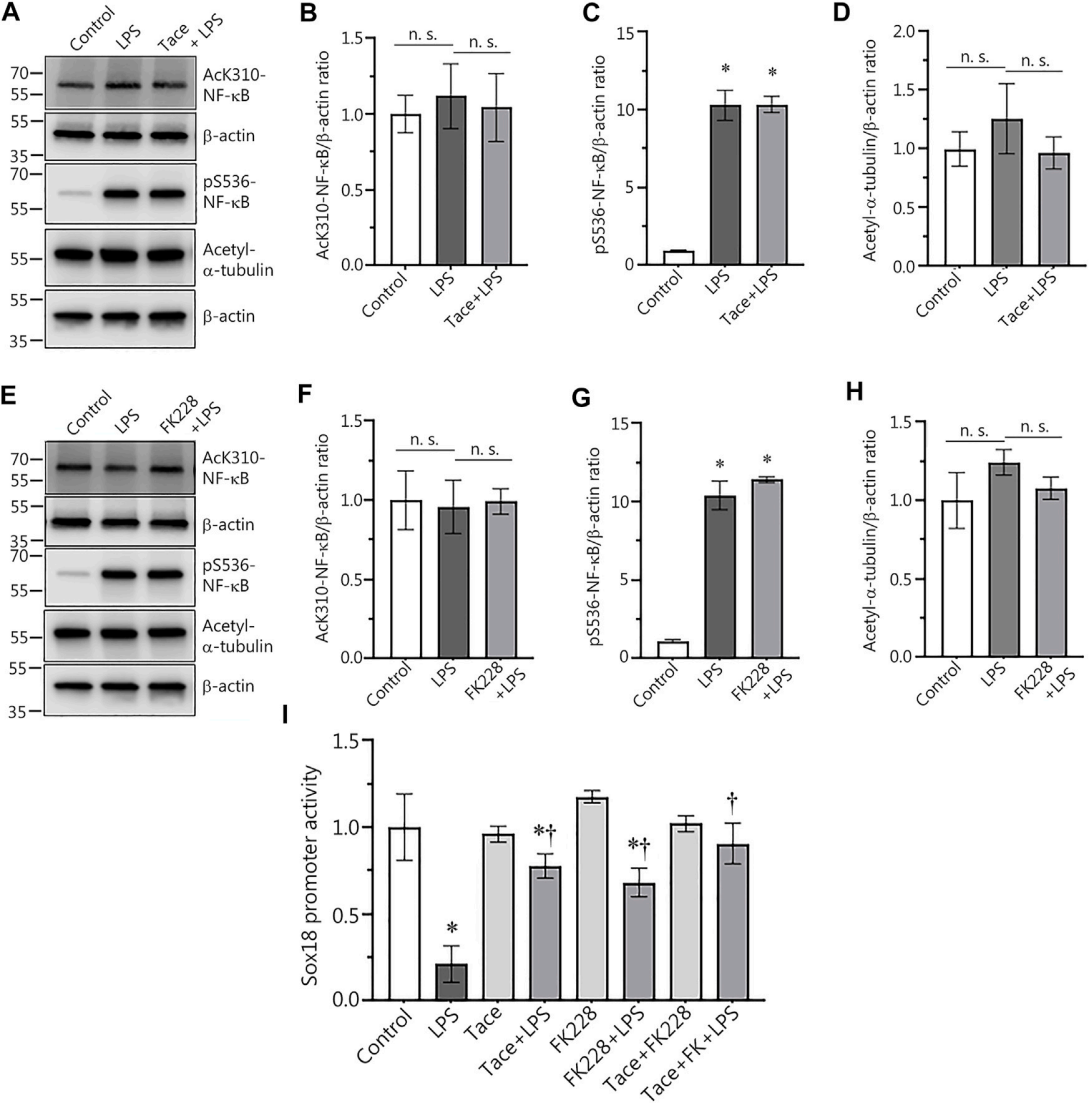
Pharmacological inhibition of HDAC1 and HDAC2 attenuates LPS-induced repression of Sox18 promoter activity in cultured human lung microvascular endothelial cells. Inhibitors of HDAC1 and HDAC2 did not affect NF-κB acetylation **(A,B,E,F)** or NF-κB phosphorylation **(A,C,E,G)** in HLMVEC. HDAC 1 or 2 inhibition did not alter the acetylation levels of the HDAC6 protein substrate, *a*-tubulin **(A,D,E,H)**. However, HDAC 1 or 2 inhibition attenuated the LPS-induced repression of the Sox18 promoter linked to a luciferase reporter **(I)**. The cells were pre-treated with the inhibitors or vehicle for 30 min then challenged with LPS for 1 h **(A–H)** or 5 h **(I)**. Data are mean ± SEM, *n* = 3–6. *p < 0.05 *vs*. Control; †*p* < 0.05 vs. LPS alone.

Previously we demonstrated that LPS-mediated downregulation of Sox18 expression in HLMVEC is NF-κB-dependent (Gross et al., 2018). Thus, we could not exclude the possibility that the protective effect on Sox18 expression with HDAC1/2 inhibitors was associated with changes in NF-κB activation status. To test this possibility, we compared NF-κB activation upon LPS stimulation in the absence or presence of HDAC1/2 inhibitors. Our data show that pre-treatment of HLMVEC with tacedinaline did not alter LPS-induced NF-κB activation as determined by evaluating p65 phosphorylation at S^536^ (Figures 3A,C) or acetylation at K^310^ levels (Figures 3A,B). As a control, we also found that Tacedinaline did not affect the acetylation of the HDAC6 substrate *a*-tubulin (Figures 3A,D) indicating that HDAC6 activity was not affected and that the barrier-protective effects of HDAC1/2 inhibition did not interfere with HDAC6. Similarly, LPS-mediated increases in LPS-induced p65 phosphorylation at S^536^ (Figures 3E,G) or acetylation at K^310^ levels (Figures 3E,F) was unaffected by FK228. Again, the acetylation of *a*-tubulin was unaffected (Figures 3E,H). To further investigate the link between HDAC1/2 and the NF-κB-dependent downregulation of Sox18 expression, we evaluated the effects of these inhibitors on SOX18 promoter activity using the Sox18-promoter luciferase construct we have previously described (Gross et al., 2018). HLMVEC were transfected with this construct and changes in luciferase levels were evaluated in LPS-challenged HLMVEC in the presence or absence of the HDAC1 or HDAC2 inhibitors. While LPS treatment alone dramatically decreased luciferase activity (Figure 3I), pre-treatment with either tacedinaline (Figure 3I) or FK228 (Figure 3I) significantly preserved luciferase activity indicating that HDAC1 and HDAC2 negatively regulate Sox18 expression *via* repression of its promoter activity.

### Silencing HDAC1 or HDAC2 expression has a positive effect on Sox18 promoter activity in LPS-challenged human lung microvascular endothelial cells

**FIGURE 4:**
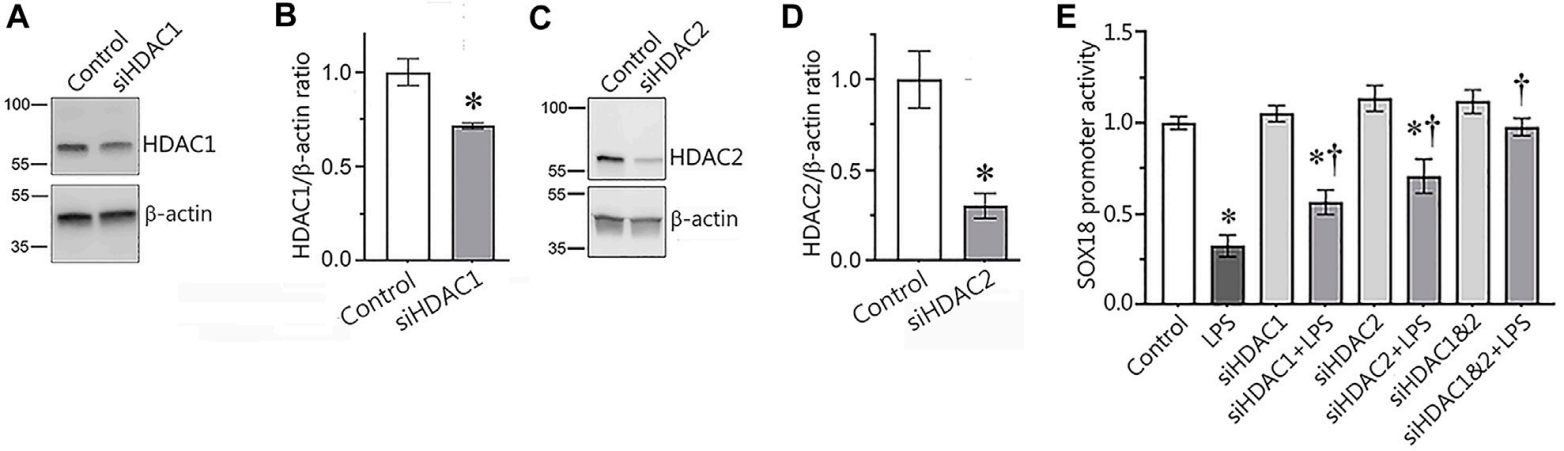
siRNA-mediated depletion of HDAC1 or HDAC2 attenuates the LPS-induced repression of the Sox18 promoter in cultured human lung microvascular endothelial cells. HLMVEC were transfected with scrambled-, HDAC1- **(A,B)**, or HDAC2- **(C,D)** siRNAs and Western blot analysis was used to confirm mRNA depletion. HDAC1 or HDAC2 depletion attenuated the LPS-mediated repression of a SOX18-luciferase construct **(E)**. Data are mean ± SEM, *n* = 3 (for immunoblots), *n* = 9–12 (for luciferase assay). *p < 0.05 *vs*. Control; †*p* < 0.05 *vs*. LPS alone.

In our next set of experiments, we used a molecular approach to evaluate the involvement of HDAC1/2 in the regulation of Sox18 expression. To accomplish this, we depleted HDAC1 (Figures 4A,B) or HDAC2 (Figures 4C,D) expression in HLMVEC using specific siRNAs and evaluated the effects on Sox18 promoter activity when HLMVEC were transfected with luciferase gene under Sox18 promoter in the presence or absence of LPS. As a negative control, HLMVEC were transfected with a scrambled siRNA. Our data show that the LPS-mediated decrease in Sox18 promoter activity (Figure 4E) is attenuated with siRNA-directed depletion of HDAC1 or HDAC2 either alone or in combination (Figure 4E) with a trend towards additive preservation (Figure 4E).

### HDAC1 associates with the NF-κB-binding site in the Sox18 promoter in LPS-challenged human lung microvascular endothelial cells

**FIGURE 5:**
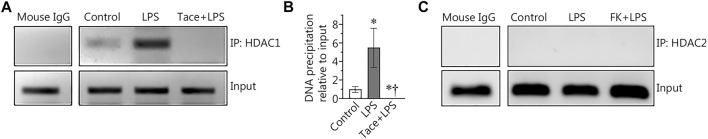
Chromatin immunoprecipitation (ChIP) analysis of HDAC1 and HDAC2 in LPS treated human lung microvascular endothelial cells. LPS increased the association of HDAC1 with the NF-κB-binding site 1 (−1082 to −1073 bp) within the Sox18 promoter sequence, and this was prevented by tacedinaline pre-treatment **(A,B)**. The association of HDAC2 with the NF-κB-binding site 1 was not detected in HLMVEC in the presence or absence of LPS **(C)**. Data are mean ± SEM, *n* = 3–5. *p < 0.05 *vs*. Control; †*p* < 0.05 *vs*. LPS alone.

To further explore the link between the HDAC1/2-dependent negative effects on Sox18 gene expression and the repressive function of NF-κB, we next utilized chromatin immunoprecipitation (ChIP) to investigate whether HDAC1 and/or HDAC2 could directly bind to the NF-κB-binding site we have previously identified in the Sox18 promoter (Gross et al., 2018). Our data show that HDAC1 associates with the NF-κB-binding site in the Sox18 promoter and this is dramatically increased upon LPS stimulation (Figures 5A,B). However, pre-treatment with the HDAC1 inhibitor, tacedinaline, completely abolished the association of HDAC1 with the NF-κB-binding site in the Sox18 promoter (Figures 5A,B). However, a similar assay performed examining HDAC2 did not reveal any association with the NF-κB-binding site in the Sox18 promoter of the Sox18 promoter region in any experimental condition (Figure 5C). These data suggest that HDAC1 is responsible for the initial binding to the promoter and then acts as a scaffold for the binding of HDAC2.

### HDAC1 inhibition preserves SOX18 expression and reduces injury in and LPS-model of acute lung injury

**FIGURE 6:**
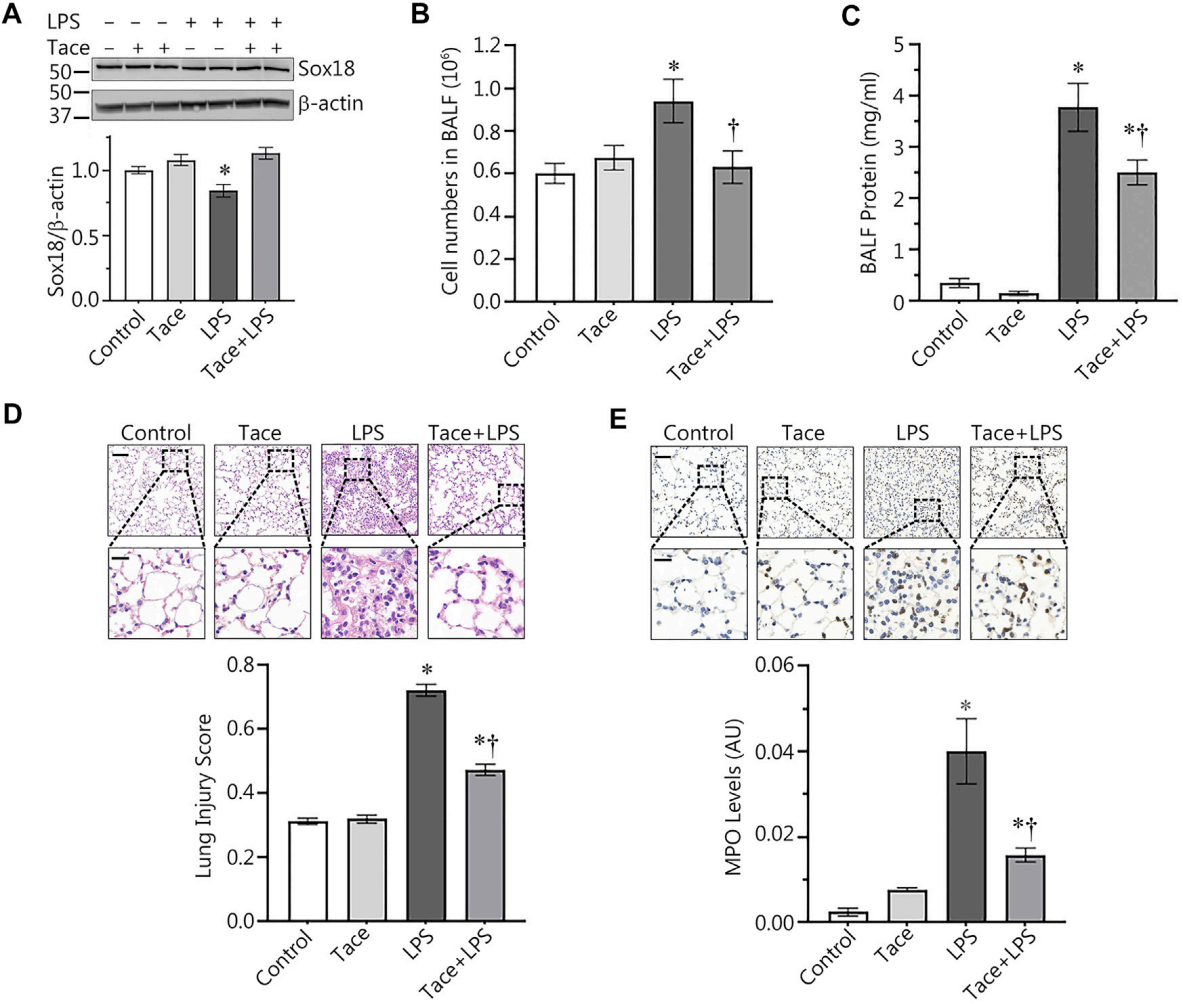
HDAC1 inhibition attenuates ALI in the mouse. Mice were pretreated with tacedinaline before being exposed to LPS. Pharmacological inhibition of HDAC1 by tacedinaline preserves Sox18 protein levels **(A)** and protects the pulmonary endothelial barrier as demonstrated by decreases in the number of inflammatory cells **(B)** and protein concentration **(C)** in the BALF. Lung sections were H&E stained, examined for signs of inflammation **(D)**, representative images shown) and scored for lung injury. The mice treated with tacedinaline had a lower lung injury score. Myeloperoxidase (MPO) immunostaining of the lung sections demonstrated a presence of activated neutrophils in the lungs exposed to LPS. Tacedinaline pre-treatment significantly decreased a number of neutrophils **(E)**. Data are mean ± SEM, *n* = 4–12. *p < 0.05 *vs*. Control; †*p* < 0.05 *vs*. LPS alone.

Our discovery of the barrier-protective effects of HDAC1/2 inhibition in LPS-challenged HLMVEC monolayers led us to test this effect *in vivo*. To accomplish this, we evaluated protective effects of tacedinaline, in a mouse model of ALI induced through intratracheal instillation of LPS. As we have previously shown (Gross et al., 2018), LPS treatment significantly decreased Sox18 protein levels in the mouse lung (Figure 6A). However, tacedinaline pre-treatment was able to significantly preserve Sox18 protein levels in the lungs of LPS-challenged mice (Figure 6A). In addition, we were able to demonstrate that the LPS-mediated increase in cellular infiltration in the bronchoalveolar lavage (BALF, Figure 6B) as well as the total protein concentration in BALF samples (Figure 6C) were significantly reduced by tacedinaline pre-treatment (Figures 6B,C) indicating a reduction in vascular hyperpermeability. As shown in H&E-stained lung sections, tacedinaline protected the lungs against LPS-induced damage and histopathological changes (Figure 6D). We assessed the lung injury severity using a semi-quantitative histopathological scoring system (Matute-Bello et al., 2001), which determines the thickness of alveolar septae, alveolar hemorrhage, intra-alveolar fibrin accumulation, and intra-alveolar infiltration. Tacedinaline pre-treatment attenuated the lung injury score in the LPS-treated mice (Figure 6D). In addition, we performed myeloperoxidase (MPO) immunostaining of the lung sections (Figure 6E) to demonstrate a presence of activated neutrophils in the lungs exposed to LPS. Tacedinaline pre-treatment significantly decreased MPO staining intensity and, therefore, a number of neutrophils in the lungs of LPS-challenged mice (Figure 6E). Therefore, we can conclude that the inhibition of HDAC1 in an animal model of ALI has a prominent therapeutic effect associated with the preservation of Sox18 expression.

## Discussion

Epigenetic regulation of gene expression plays a central role in the processes of cell and tissue functions under both physiological and pathological conditions (Yan et al., 2010; Bauer and Martin, 2017; Zarzour et al., 2019; Pagiatakis et al., 2021). Histone deacetylases (HDACs) are the enzymes responsible for chromatin inactivation *via* the removal of acetyl residues from acetylated lysines of histones (De Ruijter et al., 2003). This enhances DNA/histone interactions and, therefore, act as transcription repressors (De Ruijter et al., 2003). A growing body of evidence has documented a role for HDACs as epigenetic regulators in various pathologies. For example, a critical role for HDAC1 and 2 in carcinogenesis has been demonstrated (Li and Zhu, 2014). Thus, it is likely that a complex pathology such as ALI/ARDS is regulated, at least in part, by HDACs, since pulmonary cells *in vitro* and lung tissue *in vivo* exposed to ALI/ARDS stimuli exhibit global changes in gene transcription patterns (Dos Santos et al., 2004; Dos Santos et al., 2008; Lynn et al., 2019). Using several lines of evidence, we show here that nuclear HDACs 1 and 2 are critically involved in the endothelial barrier disruption associated with ALI. The work we report here adds important new information to the field by showing that HDACs 1 and 2 can act, at least in part, through the NF-κB-mediated repression of Sox18 gene expression. Furthermore, we were able to demonstrate for the first time, significant protective effects of the selective HDAC1 inhibitor, tacedinaline in an LPS-induced mouse model of ALI. Further, we show that tacedinaline preserves the endothelial barrier, at least in part, by preserving Sox18 expression. As we have recently demonstrated that expression of the endothelial TJ protein, claudin-5, which is critical for the integrity of pulmonary endothelium *in vitro* and *in vivo* (Gross et al., 2014; Gross et al., 2018) is regulated by Sox18 (Gross et al., 2014; Gross et al., 2018) this is likely the downstream mediator.

Sox18 belongs to the F family of Sox transcription factors (Dunn et al., 1995; Kanai et al., 1996; Taniguchi et al., 1999). In mice, Sox18 is expressed mainly in lung, heart, and skeletal muscle (Dunn et al., 1995; Hosking et al., 2001). Human Sox18 can be detected in adult and fetal tissues including brain, heart, skeletal muscle, spleen, kidney, liver, and lung and has been localized to the long arm of chromosome 20 (20q13.3) (Azuma et al., 2000; Pennisi et al., 2000; Stanojcic and Stevanovic, 2000). In addition to an N-terminal HMG domain which binds to 5′-AACAAAG-3′ sequence (Hosking et al., 1995), Sox18 contains a central transactivating domain capable of directly activating transcription *in vitro* (Hosking et al., 1995) and a C-terminal region which also contains a nine amino acid transactivation domain (Sandholzer et al., 2007) and mediates protein-protein interactions with partner co-activators. Decreased expression of Sox18 is characteristic for ALI models and has been directly associated with the endothelial hyperpermeability (Gross et al., 2018) while overexpression of Sox18 preserves endothelial integrity and reduced injury in the mouse lung exposed to LPS (Gross et al., 2018). Our previous studies have shown that Sox18 depletion impairs barrier function (Gross et al., 2014). Further, down-regulation of Sox18 in LPS-challenged HLMVEC is accompanied by a decrease of Claudin-5 and ZO-1 levels that is associated with intercellular gap formation (Gross et al., 2018). siRNA-mediated depletion of Claudin-5 also causes ZO-1 and VE-cadherin loss as well as gap formation in EC monolayers (Gross et al., 2014). Sox18 overexpression restores Claudin-5 levels and preserves EC monolayer integrity in TER assays, while Sox18 depletion reduces Claudin-5 expression and disrupts the EC monolayer (Fontijn et al., 2008; Gross et al., 2018).

NF-κB is well-established as an activator of pro-inflammatory gene expression under various stimuli (Verma et al., 1995; Ghosh et al., 1998). However, a less well studied role of NF-κB is as a transcription repressor (Kouba et al., 1999; Campbell et al., 2004; Shaw et al., 2006). We previously identified a specific NF-κB-binding site in Sox18 promoter region that is both essential and sufficient for NF-κB-mediated transcriptional repression of Sox18 (Gross et al., 2018). To repress transcription of certain genes, previous work has indicated that NF-κB subunits act in concert with several other proteins including the class I HDACs (Ashburner et al., 2001). A number of studies in the last two decades have revealed a complex multileveled modulation of ALI/ARDS pathological mechanism including global epigenetic regulation of the gene expression (reviewed in (Bossardi Ramos and Adam, 2021)). Previously, the barrier-disruptive effect of cytoplasmic class II HDAC6 has been studied in detail (Joshi et al., 2015; Yu et al., 2016a; Borgas et al., 2016; Yu et al., 2016b) demonstrating the involvement of this enzyme in a number of critical events such as the deacetylation (destabilizing) of microtubules (Borgas et al., 2016; Karki et al., 2019), the induction of TNF-α along with caspase-3 activation (Yu et al., 2016a; Yu et al., 2016b), and the regulation of Hsp90 function (Joshi et al., 2015). However, there has been minimal investigations into the role of HDACs related to the transcription repression events that occur in ALI/ARDS. Thus, the work we present here is important as it defines for the first time the role of HDAC1 and 2 in the NF-κB transcriptional complex involved in the repression of Sox18 expression during ALI.

Class I HDACs are localized within the nucleus and are typically found in either the Sin3 complex or the Mi-2/NuRD complex (Knoepfler and Eisenman, 1999). HDACs also repress transcription interacting with sequence-specific transcription factors directly (Ashburner et al., 2001). Indeed, NF-κB(p65)-HDAC1 complex can repress gene expression (Ashburner et al., 2001; Zhong et al., 2002). Also, p50 is able to directly interact with HDAC1 (Zhong et al., 2002), HDAC2 is associated with to NF-κB(p65)-HDAC1 complex *via* HDAC1 binding (Ashburner et al., 2001). Importantly, PKA-dependent phosphorylation of p65 subunit serves as a molecular switch lowering p65 affinity to HDAC1 and increasing its interaction with CBP/p300 (Zhong et al., 2002) for strong activation of certain gene transcription (Vanden Berghe et al., 1999). Our further identification of NF-κB/HDAC1/2 complexes associated with NF-κB-binding site of Sox18 promoter using ChIP assays revealed LPS-induced HDAC1 recruitment to this promoter site, whereas HDAC2 association was not detected. These data are in agreement with published studies demonstrated an indirect association of HDAC2 with transcription factors *via* binding to HDAC1 (Ashburner et al., 2001), although, for example, transcription repression by YY1 transcription factor *via* direct interaction with HDAC2 has also been demonstrated (Yang et al., 1996).

To evaluate a possible therapeutic effect of HDAC1 inhibition *in vivo*, we employed mouse model of ALI with intratracheal instillation of *E. coli* LPS widely used in ALI/ARDS studies. We found that tacedinaline pre-treatment preserved Sox18 protein levels in the lungs of LPS-challenged mice and protected pulmonary endothelium integrity *in vivo*. Moreover, morphological study of lungs obtained from mice exposed to LPS as well as MPO levels as a function of activated neutrophils clearly demonstrated lung-protective effects and a decrease in inflammation associated with tacedinaline. Therefore, we can conclude that transcription repressors HDAC1 and HDAC2 involve in molecular mechanism of ALI, their functions are associated with NF-κB-dependent down-regulation of Sox18, transcription factor critical for pulmonary endothelium integrity. Inhibition of HDAC1 has an endothelial-protective effect in pre-clinical model of ALI, and compounds with selective HDAC1/2 inhibitory properties may have a therapeutic potential in ALI/ARDS treatment.

## Data Availability

The raw data supporting the conclusion of this article will be made available by the authors, without undue reservation.

## References

[B1] Ashburner B. P. Westerheide S. D. Baldwin A. S. JR. (2001). The p65 (RelA) subunit of NF-kappaB interacts with the histone deacetylase (HDAC) corepressors HDAC1 and HDAC2 to negatively regulate gene expression. Mol. Cell. Biol. 21, 7065–7077. 10.1128/MCB.21.20.7065-7077.2001 11564889 PMC99882

[B2] Azuma T. Seki N. Yoshikawa T. Saito T. Masuho Y. Muramatsu M. (2000). cDNA cloning, tissue expression, and chromosome mapping of human homolog of SOX18. J. Hum. Genet. 45, 192–195. 10.1007/s100380050210 10807548

[B3] Baetz D. Shaw J. Kirshenbaum L. A. (2005). Nuclear factor-kappaB decoys suppress endotoxin-induced lung injury. Mol. Pharmacol. 67, 977–979. 10.1124/mol.105.011296 15673601

[B4] Bauer A. J. Martin K. A. (2017). Coordinating regulation of gene expression in cardiovascular disease: interactions between chromatin modifiers and transcription factors. Front. Cardiovasc. Med. 4, 19. 10.3389/fcvm.2017.00019 28428957 PMC5382160

[B5] Borgas D. Chambers E. Newton J. Ko J. Rivera S. Rounds S. (2016). Cigarette smoke disrupted lung endothelial barrier integrity and increased susceptibility to acute lung injury *via* histone deacetylase 6. Am. J. Respir. Cell Mol. Biol. 54, 683–696. 10.1165/rcmb.2015-0149OC 26452072 PMC4942194

[B6] Bossardi Ramos R. Adam A. P. (2021). Molecular mechanisms of vascular damage during lung injury. Adv. Exp. Med. Biol. 1304, 95–107. 10.1007/978-3-030-68748-9_6 34019265 PMC8223730

[B7] Campbell K. J. Rocha S. Perkins N. D. (2004). Active repression of antiapoptotic gene expression by RelA(p65) NF-kappa B. Mol. Cell 13, 853–865. 10.1016/s1097-2765(04)00131-5 15053878

[B8] De Ruijter A. J. Van Gennip A. H. Caron H. N. Kemp S. Van Kuilenburg A. B. (2003). Histone deacetylases (HDACs): Characterization of the classical HDAC family. Biochem. J. 370, 737–749. 10.1042/BJ20021321 12429021 PMC1223209

[B9] Dos Santos C. C. Han B. Andrade C. F. Bai X. Uhlig S. Hubmayr R. (2004). DNA microarray analysis of gene expression in alveolar epithelial cells in response to TNFalpha, LPS, and cyclic stretch. Physiol. Genomics 19, 331–342. 10.1152/physiolgenomics.00153.2004 15454581

[B10] Dos Santos C. C. Okutani D. Hu P. Han B. Crimi E. He X. (2008). Differential gene profiling in acute lung injury identifies injury-specific gene expression. Crit. Care Med. 36, 855–865. 10.1097/CCM.0B013E3181659333 18431273

[B11] Dunn T. L. Mynett-Johnson L. Wright E. M. Hosking B. M. Koopman P. A. Muscat G. E. (1995). Sequence and expression of Sox-18 encoding a new HMG-box transcription factor. Gene 161, 223–225. 10.1016/0378-1119(95)00280-j 7665083

[B12] Fiordelisi A. Iaccarino G. Morisco C. Coscioni E. Sorriento D. (2019). NFkappaB is a key player in the crosstalk between inflammation and cardiovascular diseases. Int. J. Mol. Sci. 20, E1599. 10.3390/ijms20071599 30935055 PMC6480579

[B13] Fontijn R. D. Volger O. L. Fledderus J. O. Reijerkerk A. De Vries H. E. Horrevoets A. J. (2008). SOX-18 controls endothelial-specific claudin-5 gene expression and barrier function. Am. J. Physiol. Heart Circ. Physiol. 294, H891–H900. 10.1152/ajpheart.01248.2007 18065521

[B14] Garcia J. G. Schaphorst K. L. Verin A. D. Vepa S. Patterson C. E. Natarajan V. (2000). Diperoxovanadate alters endothelial cell focal contacts and barrier function: role of tyrosine phosphorylation. J. Appl. Physiol. (1985) 89, 2333–2343. 10.1152/jappl.2000.89.6.2333 11090587

[B15] Ghosh S. May M. J. Kopp E. B. (1998). NF-Kappa B and rel proteins: Evolutionarily conserved mediators of immune responses. Annu. Rev. Immunol. 16, 225–260. 10.1146/annurev.immunol.16.1.225 9597130

[B16] Gross C. M. Aggarwal S. Kumar S. Tian J. Kasa A. Bogatcheva N. (2014). Sox18 preserves the pulmonary endothelial barrier under conditions of increased shear stress. J. Cell. Physiol. 229, 1802–1816. 10.1002/jcp.24633 24677020 PMC4117725

[B17] Gross C. M. Kellner M. Wang T. Lu Q. Sun X. Zemskov E. A. (2018). LPS-Induced acute lung injury involves NF-κB-mediated downregulation of SOX18. Am. J. Respir. Cell Mol. Biol. 58, 614–624. 10.1165/rcmb.2016-0390OC 29115856 PMC5946326

[B18] Hosking B. M. Muscat G. E. Koopman P. A. Dowhan D. H. Dunn T. L. (1995). Trans-activation and DNA-binding properties of the transcription factor, Sox-18. Nucleic Acids Res. 23, 2626–2628. 10.1093/nar/23.14.2626 7651823 PMC307084

[B19] Hosking B. M. Wyeth J. R. Pennisi D. J. Wang S. C. Koopman P. Muscat G. E. (2001). Cloning and functional analysis of the Sry-related HMG box gene, Sox18. Gene 262, 239–247. 10.1016/s0378-1119(00)00525-4 11179689

[B20] Joshi A. D. Barabutis N. Birmpas C. Dimitropoulou C. Thangjam G. Cherian-Shaw M. (2015). Histone deacetylase inhibitors prevent pulmonary endothelial hyperpermeability and acute lung injury by regulating heat shock protein 90 function. Am. J. Physiol. Lung Cell. Mol. Physiol. 309, L1410–L1419. 10.1152/ajplung.00180.2015 26498249 PMC4683315

[B21] Kanai Y. Kanai-Azuma M. Noce T. Saido T. C. Shiroishi T. Hayashi Y. (1996). Identification of two Sox17 messenger RNA isoforms, with and without the high mobility group box region, and their differential expression in mouse spermatogenesis. J. Cell Biol. 133, 667–681. 10.1083/jcb.133.3.667 8636240 PMC2120827

[B22] Karki P. Meliton A. Sitikov A. Tian Y. Ohmura T. Birukova A. A. (2019). Microtubule destabilization caused by particulate matter contributes to lung endothelial barrier dysfunction and inflammation. Cell. Signal. 53, 246–255. 10.1016/j.cellsig.2018.10.010 30339829 PMC6553475

[B23] Knoepfler P. S. Eisenman R. N. (1999). Sin meets NuRD and other tails of repression. Cell 99, 447–450. 10.1016/s0092-8674(00)81531-7 10589671

[B24] Kouba D. J. Chung K. Y. Nishiyama T. Vindevoghel L. Kon A. Klement J. F. (1999). Nuclear factor-kappa B mediates TNF-alpha inhibitory effect on alpha 2(I) collagen (COL1A2) gene transcription in human dermal fibroblasts. J. Immunol. 162, 4226–4234. 10201951

[B25] Kumar S. Sun X. Wedgwood S. Black S. M. (2008). Hydrogen peroxide decreases endothelial nitric oxide synthase promoter activity through the inhibition of AP-1 activity. Am. J. Physiol. Lung Cell. Mol. Physiol. 295, L370–L377. 10.1152/ajplung.90205.2008 18556800 PMC2519847

[B26] Li Z. Zhu W. G. (2014). Targeting histone deacetylases for cancer therapy: from molecular mechanisms to clinical implications. Int. J. Biol. Sci. 10, 757–770. 10.7150/ijbs.9067 25013383 PMC4081609

[B27] Lynn H. Sun X. Casanova N. Gonzales-Garay M. Bime C. Garcia J. G. N. (2019). Genomic and genetic approaches to deciphering acute respiratory distress syndrome risk and mortality. Antioxid. Redox Signal. 31, 1027–1052. 10.1089/ars.2018.7701 31016989 PMC6939590

[B28] Maniatis N. A. Kotanidou A. Catravas J. D. Orfanos S. E. (2008). Endothelial pathomechanisms in acute lung injury. Vasc. Pharmacol. 49, 119–133. 10.1016/j.vph.2008.06.009 PMC7110599 18722553

[B29] Matute-Bello G. Downey G. Moore B. B. Groshong S. D. Matthay M. A. Slutsky A. S. (2011). An official American thoracic society workshop report: Features and measurements of experimental acute lung injury in animals. Am. J. Respir. Cell Mol. Biol. 44, 725–738. 10.1165/rcmb.2009-0210ST 21531958 PMC7328339

[B30] Matute-Bello G. Winn R. K. Jonas M. Chi E. Y. Martin T. R. Liles W. C. (2001). Fas (CD95) induces alveolar epithelial cell apoptosis *in vivo*: Implications for acute pulmonary inflammation. Am. J. Pathol. 158, 153–161. 10.1016/S0002-9440(10)63953-3 11141488 PMC1850249

[B31] Nitta T. Hata M. Gotoh S. Seo Y. Sasaki H. Hashimoto N. (2003). Size-selective loosening of the blood-brain barrier in claudin-5-deficient mice. J. Cell Biol. 161, 653–660. 10.1083/jcb.200302070 12743111 PMC2172943

[B32] Pagiatakis C. Musolino E. Gornati R. Bernardini G. Papait R. (2021). Epigenetics of aging and disease: a brief overview. Aging Clin. Exp. Res. 33, 737–745. 10.1007/s40520-019-01430-0 31811572 PMC8084772

[B33] Pahl H. L. (1999). Activators and target genes of Rel/NF-kappaB transcription factors. Oncogene 18, 6853–6866. 10.1038/sj.onc.1203239 10602461

[B34] Pennisi D. J. James K. M. Hosking B. Muscat G. E. Koopman P. (2000). Structure, mapping, and expression of human SOX18. Mamm. Genome 11, 1147–1149. 10.1007/s003350010216 11130989

[B35] Rafikov R. Dimitropoulou C. Aggarwal S. Kangath A. Gross C. Pardo D. (2014). Lipopolysaccharide-induced lung injury involves the nitration-mediated activation of RhoA. J. Biol. Chem. 289, 4710–4722. 10.1074/jbc.M114.547596 24398689 PMC3931033

[B36] Sandholzer J. Hoeth M. Piskacek M. Mayer H. De Martin R. (2007). A novel 9-amino-acid transactivation domain in the C-terminal part of Sox18. Biochem. Biophys. Res. Commun. 360, 370–374. 10.1016/j.bbrc.2007.06.095 17603017

[B37] Schwartz M. D. Moore E. E. Moore F. A. Shenkar R. Moine P. Haenel J. B. (1996). Nuclear factor-kappa B is activated in alveolar macrophages from patients with acute respiratory distress syndrome. Crit. Care Med. 24, 1285–1292. 10.1097/00003246-199608000-00004 8706481

[B38] Shaw J. Zhang T. Rzeszutek M. Yurkova N. Baetz D. Davie J. R. (2006). Transcriptional silencing of the death gene BNIP3 by cooperative action of NF-kappaB and histone deacetylase 1 in ventricular myocytes. Circ. Res. 99, 1347–1354. 10.1161/01.RES.0000251744.06138.50 17082476

[B39] Stanojcic S. Stevanovic M. (2000). The human SOX18 gene: cDNA cloning and high resolution mapping. Biochim. Biophys. Acta 1492, 237–241. 10.1016/s0167-4781(00)00078-6 10858556

[B40] Taniguchi K. Hiraoka Y. Ogawa M. Sakai Y. Kido S. Aiso S. (1999). Isolation and characterization of a mouse SRY-related cDNA, mSox7. Biochim. Biophys. Acta 1445, 225–231. 10.1016/s0167-4781(99)00047-0 10320775

[B41] Vanden Berghe W. De Bosscher K. Boone E. Plaisance S. Haegeman G. (1999). The nuclear factor-kappaB engages CBP/p300 and histone acetyltransferase activity for transcriptional activation of the interleukin-6 gene promoter. J. Biol. Chem. 274, 32091–32098. 10.1074/jbc.274.45.32091 10542243

[B42] Verma I. M. Stevenson J. K. Schwarz E. M. Van Antwerp D. Miyamoto S. (1995). Rel/NF-kappa B/I kappa B family: Intimate tales of association and dissociation. Genes Dev. 9, 2723–2735. 10.1101/gad.9.22.2723 7590248

[B43] Yan M. S. Matouk C. C. Marsden P. A. (2010). Epigenetics of the vascular endothelium. J. Appl. Physiol. (1985) 109, 916–926. 10.1152/japplphysiol.00131.2010 20413423

[B44] Yang W. M. Inouye C. Zeng Y. Bearss D. Seto E. (1996). Transcriptional repression by YY1 is mediated by interaction with a mammalian homolog of the yeast global regulator RPD3. Proc. Natl. Acad. Sci. U. S. A. 93, 12845–12850. 10.1073/pnas.93.23.12845 8917507 PMC24008

[B45] Yu J. Ma M. Ma Z. Fu J. (2016a). HDAC6 inhibition prevents TNF-alpha-induced caspase 3 activation in lung endothelial cell and maintains cell-cell junctions. Oncotarget 7, 54714–54722. 10.18632/oncotarget.10591 27419634 PMC5342375

[B46] Yu J. Ma Z. Shetty S. Ma M. Fu J. (2016b). Selective HDAC6 inhibition prevents TNF-alpha-induced lung endothelial cell barrier disruption and endotoxin-induced pulmonary edema. Am. J. Physiol. Lung Cell. Mol. Physiol. 311, L39–L47. 10.1152/ajplung.00051.2016 27190059

[B47] Zarzour A. Kim H. W. Weintraub N. L. (2019). Epigenetic regulation of vascular diseases. Arterioscler. Thromb. Vasc. Biol. 39, 984–990. 10.1161/ATVBAHA.119.312193 31070469 PMC6531339

[B48] Zhong H. May M. J. Jimi E. Ghosh S. (2002). The phosphorylation status of nuclear NF-kappa B determines its association with CBP/p300 or HDAC-1. Mol. Cell 9, 625–636. 10.1016/s1097-2765(02)00477-x 11931769

